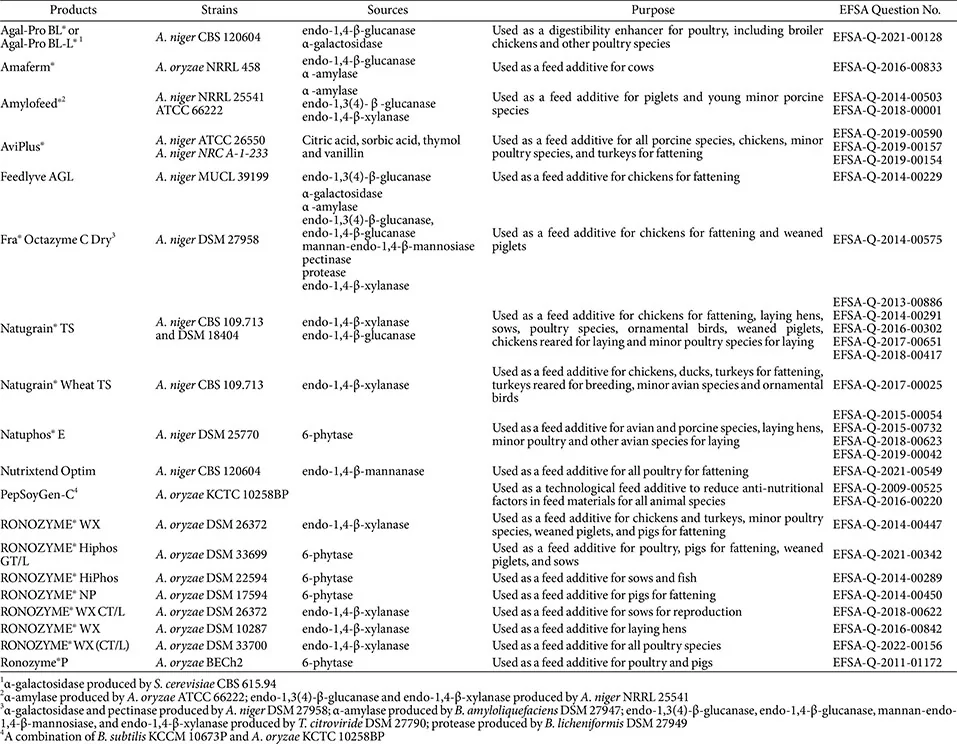# Applications of Fungi and Fungal-Derived Ingredients in the QPS and GRAS Systems

**DOI:** 10.4014/jmb.2607.07007

**Published:** 2026-08-20

**Authors:** He-Jin Cho, Yeon Ju Lee, Young Hoon Jung, Hee-Soo Park

**Affiliations:** 1 School of Food Science and Biotechnology, Kyungpook National University, Daegu 41566, Republic of Korea; 2 Department of Food Science and Biotechnology, Universitas Brawijaya, Malang 65145, Indonesia; 3 Department of Integrative Biology, Kyungpook National University, Daegu 41566, Republic of Korea; 4 Department of Advanced Bioconvergence, Kyungpook National University, Daegu 41566, Republic of Korea

**Keywords:** GRAS, QPS, *Aspergillus oryzae*, *Aspergillus niger*

## Abstract

Filamentous fungi are representative eukaryotic microorganisms that are widely used in the food industry for the production of organic acids, enzymes, minerals, and other value-added compounds. However, ensuring their safety is a prerequisite for their application in food production. Two major regulatory approaches relevant to the safety of microbial food production are the U.S. Food and Drug Administration (FDA) Generally Recognized as Safe (GRAS) framework and the European Food Safety Authority (EFSA) Qualified Presumption of Safety (QPS) approach. The EFSA QPS framework evaluates eligible taxonomic units, whereas FDA GRAS Notices concern specified substances produced under defined conditions, including substances manufactured using yeasts, filamentous fungi, and mushrooms. Among these, several species of filamentous fungi, particularly members of the genus *Aspergillus*, have been widely used for the industrial production of food enzymes and other commercially important bioproducts. Notably, selected strains of *Aspergillus oryzae* and *Aspergillus niger* have a long history of industrial use in the production of enzymes, organic acids, and other industrial products. Enzymes produced by *A. oryzae* and *A. niger* are used in food processing as well as in animal feed to improve animal nutrition and digestion. This review provides a comprehensive overview of the *Aspergillus* taxa evaluated under the QPS approach and GRAS Notices involving *Aspergillus* production organisms or derived substances and discusses their current and potential applications in the food industry.

## Introduction

Fungi include molds, yeasts, and mushrooms, which are eukaryotic organisms that can exist in either unicellular or multicellular forms [1, 2]. They are characterized by the presence of vacuole-like structures and the absence of chloroplasts [3]. Additionally, although fungi possess cell walls, their cell walls are primarily composed of chitin, which distinguishes them from plant cells [4, 5]. Fungi obtain nutrients by secreting various enzymes that degrade macromolecules into smaller molecules, which allows them to survive in different environments [6]. Consequently, fungi play an essential role as decomposers in ecosystems [2, 7]. These characteristics of fungi have also been widely applied in the food industry, significantly contributing to the development of food science [8]. In East Asia, various fungi, such as *Aspergillus oryzae*, *Rhizopus* spp., and *Mucor* spp., are involved in the processing of traditional fermented foods, contributing to their taste, flavor, and quality [9, 10]. In the cheese and brewing industries, various fungal species, including *Penicillium* spp. and *Saccharomyces* spp., are also used in fermentation processes [11]. Fungi are directly used in food production processes, and their primary or secondary metabolites, such as enzymes and organic acids, are also widely applied in the food industry [12, 13]. For instance, a wide range of food enzymes, including lipase, amylase, and phospholipase, produced by either wild-type or genetically modified fungi, are used in various food industry applications [14, 15].

Although fungi play beneficial roles through their applications in food production, some fungal species are classified as plant or animal pathogens and can cause various diseases [16-18]. Certain fungi produce toxic secondary metabolites, referred to as mycotoxins, which are the primary causes of food poisoning and diseases associated with fungi [19, 20]. Representative mycotoxins include aflatoxins produced by *Aspergillus flavus*, ochratoxins produced by *Aspergillus* spp. and *Penicillium* spp., and zearalenone, fumonisins, and deoxynivalenol produced by *Fusarium* spp. [20, 21].

Therefore, appropriate safety evaluation is essential before fungi or fungal-derived products can be used in food and industrial applications. Two representative and internationally recognized regulatory frameworks relevant to fungal food production are the Qualified Presumption of Safety (QPS) approach developed by the European Food Safety Authority (EFSA) and the Generally Recognized as Safe (GRAS) notification program administrated by the U.S. Food and Drug Administration (FDA) [22-24]. Although both frameworks are widely used to support the safe use of microorganisms in the food industry, they differ substantially in their scope. The EFSA QPS approach evaluates the safety of defined microbial taxonomic units, whereas FDA GRAS conclusions apply to specified substances produced under defined conditions of use and should not be interpreted as conferring general GRAS status on an entire fungal species or production strain. This review provides a comprehensive overview of fungal taxa evaluated under the EFSA QPS approach and fungal production organisms or fungal-derived substances described in FDA GRAS Notices, with a particular focus on *Aspergillus* species and their current and potential applications in the food industry.

## Fungi Microorganisms in the EFSA QPS Framework

The QPS approach developed by the EFSA provides a harmonized framework for assessing the safety of microorganisms intentionally introduced into the food or feed chain [24]. Microorganisms may be notified to EFSA in connection with applications involving feed additives, food additives, food enzymes, food flavorings, novel foods, and plant protection products. During QPS assessment, EFSA considers the taxonomic identity of the microorganisms, the available body of knowledge, potential safety concerns, and the intended use. Taxonomic units that meet the relevant criteria may be recommended for QPS status, with specific qualifications where necessary. Importantly, the inventory of microorganisms notified to EFSA for QPS assessment is distinct from the list of taxonomic units recommended for QPS status. Therefore, inclusion in the notification inventory does not necessarily indicate that a microorganism has been granted or recommended for QPS status.

The QPS inventory is updated approximately every six months and is organized into eight major microbiological groups. According to the January 2026 QPS inventory, a total of 1,539 microorganism notifications were listed, including 425 notifications involving filamentous fungi and 164 involving yeasts within the microbiological groups [25]. We manually reviewed these fungal notifications at the species level, removed duplicate species, and compiled the unique filamentous fungal and yeast species presented in Table 1.

Among the notified filamentous fungi, the most extensively used genus among filamentous fungi is *Aspergillus*, which includes *A. niger*, *A. oryzae*, *A. aculeatus*, and *A. tubingensis*. Among these, *A. niger* is the most widely used species and is employed for a wide range of applications, including enzyme production, food enzyme, food supplements, technological additive, and zootechnical additives. The genus *Trichoderma*, which includes *T. reesei*, *T. citrinoviride*, and *T. atroviride*, is the second most frequently reported group of filamentous fungi. Additionally, genera such as *Penicillium*, *Rhizopus*, and *Fusarium* are primarily notified in enzyme production, food additives, and novel foods. Mushrooms, including *Trametes hirsuta*, *Lentinula edodes*, and *Antrodia camphorata*, are also categorized with filamentous fungi. Specifically, *T. hirsuta* strain AE-OR is used to produce the food enzyme laccase [26]. *L. edodes* is notified in the fermentation of pea protein [27]. *Antrodia camphorata* has been approved as a novel food in the form of freeze-dried mycelia for use as a dietary supplement [28].

Among the notified yeasts, *Saccharomyces cerevisiae* is the most extensively notified species owing to its wide range of applications. *S. cerevisiae* is employed in biomass production, in the synthesis of various enzymes, such as α-galactosidase, triacylglycerol lipase, asparaginase, and α-amylase, and as a supplement of zootechnical additives. *Yarrowia lipolytica* is primarily used for yeast biomass production. *Pichia pastoris* is used in the production of enzymes, food additives, food flavoring, and zootechnical additives. *Komagataella phaffii* (syn. *K. pastoris*, formerly *P. pastoris*) is notified to produce various enzymes and digestibility enhancers for zootechnical applications.

## Fungi and Fungal-Derived Substances in the GRAS Notification System

In the United States, intentionally added substances in foods are regulated as food additives under the Federal Food, Drug, and Cosmetic Act (FFDCA) and require FDA approval. By contrast, substances with a long history of safe use or those deemed safe by experts are regulated under the GRAS system, which was first introduced in 1958 and implemented as a notification program in 1997 [22]. As of January 2026, more than 1,290 GRAS notices have been submitted, with approximately 1,020 receiving a “no questions” response from the FDA. It should be noted that a “no questions” response indicates that the FDA has no further questions regarding the notifier's GRAS conclusion under the intended conditions of use; it does not constitute FDA approval or confer general GRAS status on the production organism itself. All 1,290 publicly available GRAS Notices (as of January 2026) were systematically reviewed. The associated production organisms were identified from each notice and classified as filamentous fungi, mushrooms, or yeasts according to their scientific names. A total of 169 GRAS Notices involving fungal production organisms or fungal-derived substances were identified and included in the final dataset (Table 2). Of these, 108 notifications were related to filamentous fungi, 6 to mushrooms, and 55 to yeasts. Among the filamentous fungi, the most frequently reported genus was *Aspergillus* (48 notifications), followed by *Trichoderma*. *Aspergillus niger* is primarily associated with the production of food enzymes, and in some cases, fungal-derived ingredients such as chitosan or chitin-glucan. *A. oryzae* is also primarily related to enzyme and protein production but has additional applications, such as the production of brazzein and mineral-enriched Koji. At the species level, *T. reesei* was the most frequently represented production organism and was used predominantly for enzyme manufacture. GRAS Notices involving *Mortierella alpina* primarily concern arachidonic acid-rich oil, often referred to as fungal oil [29]. *Fusarium* spp. were associated with the production of mycoproteins and enzymes. Mushroom-derived ingredients, including β-glucan and chitosan produced from *Agaricus bisporus*, *Antrodia cinnamomea*, and *Ganoderma lucidum*. In addition, *L. edodes* has been used in the fermentation organism for the production of fermented pea and rice proteins.

Among yeasts, *S. cerevisiae* is the most widely utilized and was the most frequently represented production organism. GRAS Notices involved *S. cerevisiae* covered applications including starter culture for alcoholic beverages production, food ingredients such as β-glucan, selenium-enriched yeast, mannoproteins, and steviol glycosides, and, in some case, enzyme production. *K. phaffii* is the second most widely represented yeast and was extensively employed as a recombinant protein production host. Products described in GRAS Notices included sweet proteins such as brazzein and monellin as well as recombinant proteins including myoglobin, leghemoglobin, egg-white proteins, and β-lactoglobulin for use in alternative protein and meat applications. Other yeast species, including *Y. lipolytica*, *Papiliotrema terrestris*, *Hansenula polymorpha*, *Candida cylindracea*, and *Candida rugosa*, are also identified as production organisms in GRAS Notices involving enzymes and other protein products. These GRAS conclusions apply to the specific substance produced under the notified manufacturing processes and intended conditions of use, rather than to the microorganisms as a whole.

### Industrial Applications of *Aspergillus* spp.

**Fig. 1:**
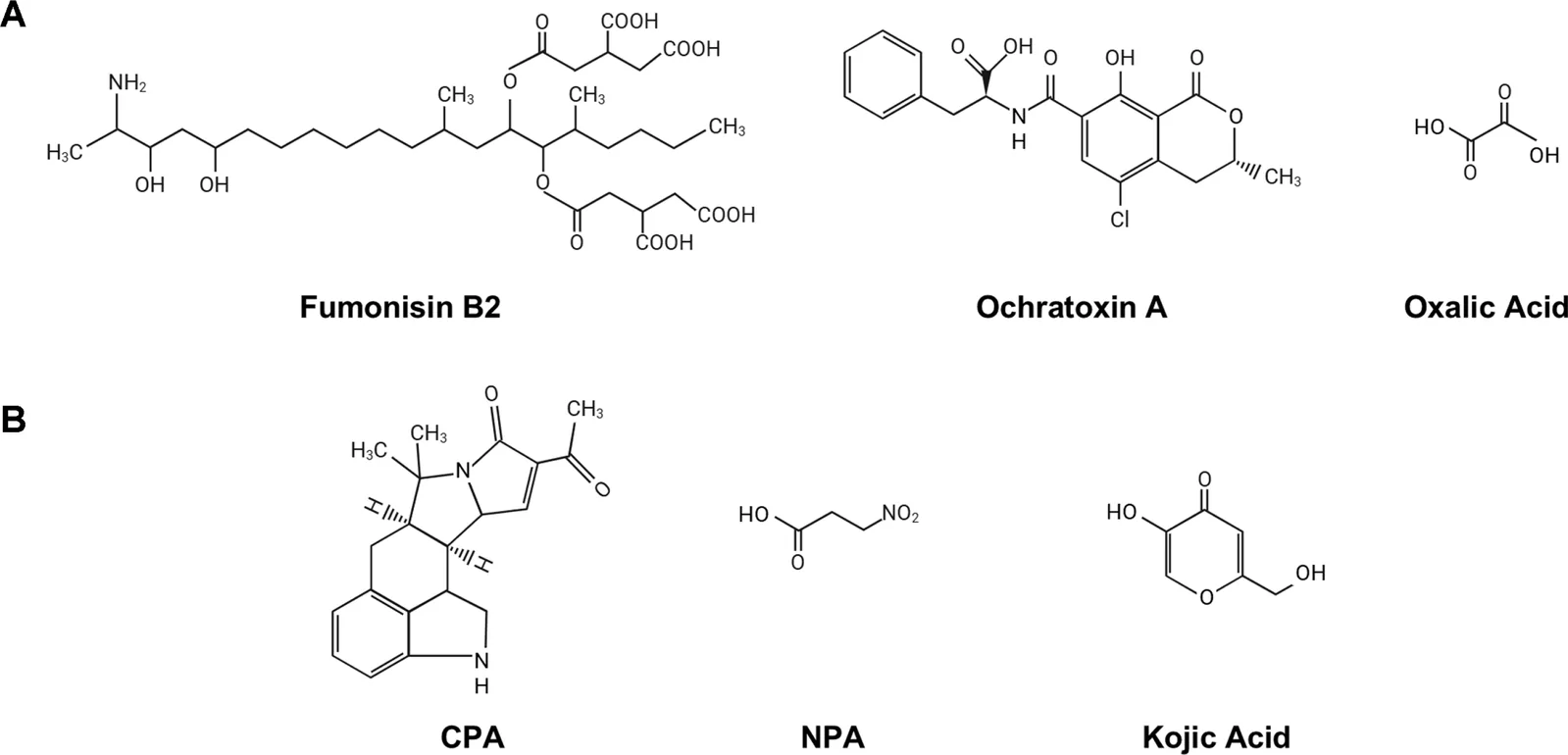
Potentially harmful secondary metabolites produced by *Aspergillus* spp. (**A**) Potentially harmful secondary metabolites reported in *A. niger*. (**B**) Potentially harmful secondary metabolites reported in *A. oryzae*, including cyclopiazonic acid (CPA) and 3-nitropropionic acid (NPA).

As mentioned above, among the fungal organisms notified to EFSA for QPS assessment and the fungal production organisms associated with FDA GRAS Notices, *Aspergillus* species account for the largest proportion. Approximately 450 species of *Aspergillus* have been identified, and these fungi are used in a wide range of fields [30]. Some species, including *A. fumigatus* and *A. flavus*, are pathogenic and can cause aspergillosis [31, 32], whereas others, such as *A. flavus* and *A. parasiticus*, are toxin-producing fungi [33]. Additionally, some secondary metabolites can have adverse effects [34, 35] (Fig. 1). Nevertheless, many *Aspergillus* species are extensively utilized in the food industry [36, 37]. As described earlier, *A. oryzae* and *A. niger* are representative fungi widely used in the food industry, and *A. tubingensis* is also utilized for enzyme production [38, 39].

### Aspergillus niger

*A. niger* is a representative fungal species belonging to the section Nigri of the genus *Aspergillus* [40]. It produces black spores and exhibits rapid growth in various natural environments [41]. *A. niger* is widely recognized as a major producer of citric acid, as well as industrially important enzymes such as amylase, protease, and pectinase [42, 43]. Selected strains of *A. niger* have been used as production organisms in GRAS Notices and have also been notified to EFSA for QPS assessment. However, these regulatory records apply to specific substances, strains, production processes, and intended conditions of use and should not be interpreted as conferring general GRAS or QPS status on the entire species [42]. Additionally, the Joint FAO/WHO Expert Committee on Food Additives (JECFA) has evaluated certain enzyme preparations produced using non-pathogenic and non-toxigenic strains of *A. niger* [44]. However, several harmful secondary metabolites produced by *A. niger*, such as ochratoxin A, fumonisins, and oxalic acid, have been reported to adversely affect human health (Fig. 1A) [45]. Therefore, the safety of each production strain and its derived product should be evaluated individually, including verification of the absence of relevant mycotoxins and other undesirable metabolites.

Among the fungal production organisms identified in GRAS Notices and EFSA notification records, *A. niger* is one of the most representative fungal species used for protein production in the food industry. According to the GRAS notification inventory, 27 GRAS Notices involved *A. niger* as a production organism (Table 3). In most cases, *A. niger* is primarily used for the production of enzymes for the food industry. It has been utilized by companies such as DSM Food Specialties, Novozymes, and Chr. Hansen (currently Novonesis). *A. niger* is also involved in the production of products other than proteins. For instance, KitoZyme utilizes *A. niger* to produce chitin and β-(1,3)-glucan (GRN 412), as well as chitosan (GRN 397). These fungal-derived substances are used in applications including the production of alcoholic beverages. The corresponding GRAS conclusions apply specifically to these notified substances under their described manufacturing processes and conditions of use, rather than to *A. niger* as a species.

**Fig. 2:**
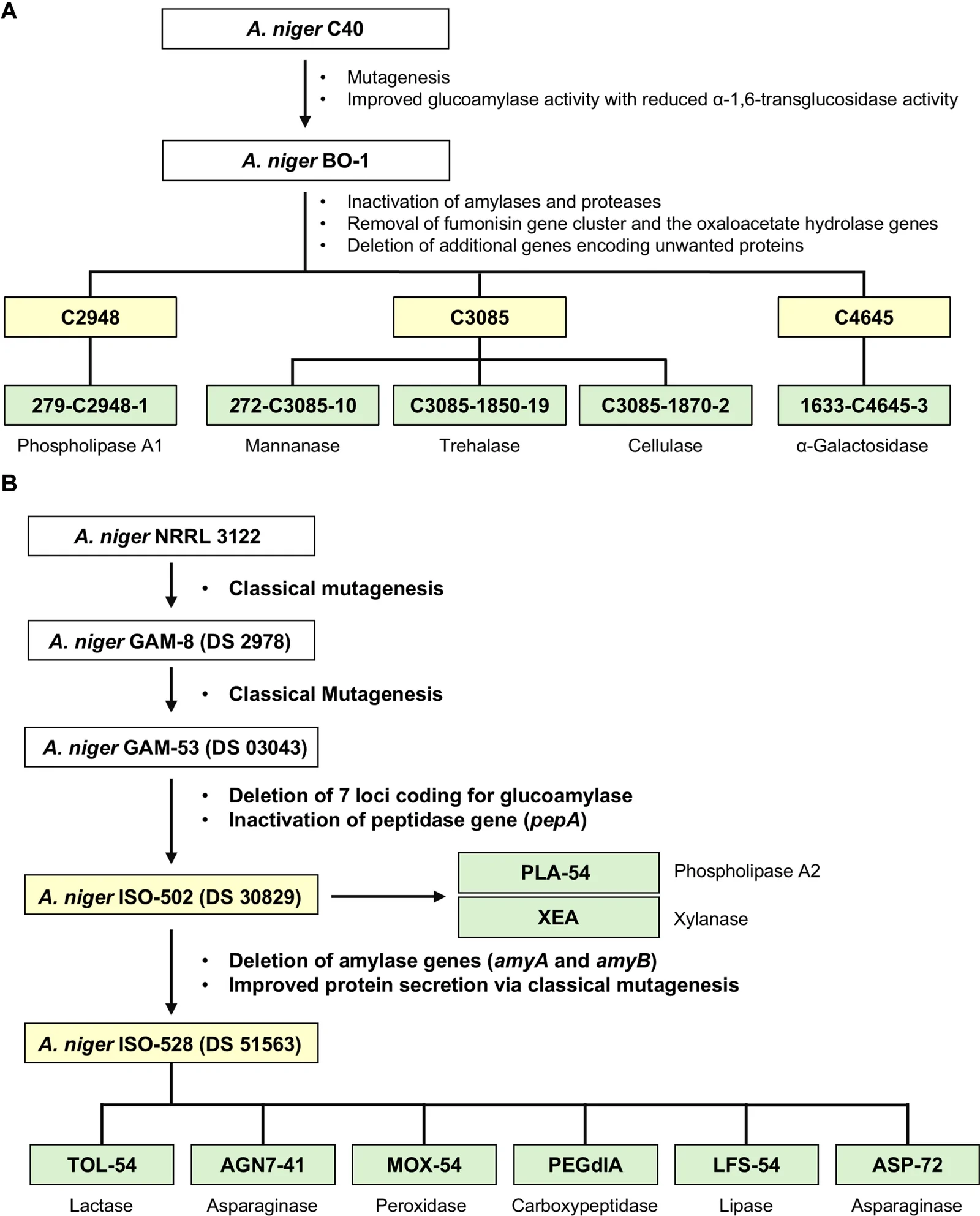
Lineages of *A. niger* strains derived from the C40 or NRRL 3122 strains. (**A**) *A. niger* C40 and its derived BO-1 lineage strains (**B**) *A. niger* NRRL 3122 and its derived lineage strains.

To produce enzymes, food companies secure and improve their own strains in order to achieve enhanced production capacity and food safety. Representative strains include *A. niger* C40 and *A. niger* NRRL 3122 (Fig. 2). First, strains used by companies such as Novozymes for protein production include *A. niger* C40 and its derived BO-1 lineage strains. *A. niger* C40 is a representative wild-type strain that was isolated from nature in the 1960s and is known as a producer of glucoamylase. This wild-type strain was improved through mutagenesis to generate the mutant strain BO-1, which showed enhanced glucoamylase production but lacked the ability to produce α-1,6-transglucosidase. Furthermore, to increase protein production efficiency and eliminate mycotoxin production, additional genetically engineered BO-1 lineage strains, such as *A. niger* C2948 (GRN 651), C3085 (GRN 699, GRN 739, and GRN 1030), and C4645 (GRN 1120), were generated. These strains were engineered by inactivating amylase and protease genes and deleting the fumonisin gene cluster and the oxaloacetate hydrolase gene. Additional genetic modification improved the efficiency and purity of protein production and simultaneously enhanced safety. These strains have been used for the production of various enzymes, such as phospholipase A1 (GRN 651), trehalase (GRN 699), mannanase (GRN 739), cellulase (GRN 1030), and α-galactosidase (GRN 1120) (Table 3).

Second, *A. niger* NRRL 3122 was improved to generate the GAM lineage, including strains such as ISO-502 and ISO-528, which have been widely used for protein production through classical mutagenesis and genetic modification. *A. niger* NRRL 3122 was sequentially edited through classical mutagenesis to produce GAM-8 (DS 2978) and subsequently GAM-53 (DS 03043). GAM-53 (DS 03043) exhibited enhanced glucoamylase production and was used in starch processing applications. *A. niger* GAM-53 was further genetically engineered by removing the glucoamylase gene to create “plug sites” and by inactivating the protease gene *pepA*, yielding strains such as ISO-502 (DS 30829), also referred to as “plug bugs.” ISO-502 was used for the expression of enzymes such as phospholipase A2 (GRN 183) and xylanase (GRN 589). Subsequently, the amylase genes *amyA* and *amyB* were deleted from ISO-502, and classical mutagenesis was further applied to generate ISO-528 (DS 51563), a strain with enhanced protein secretion capability. ISO-528 has been used for the expression of homologous and heterologous proteins to produce enzymes such as asparaginase (GRN 214), lipase (GRN 296), carboxypeptidase (GRN 345), peroxidase (GRN 402), asparaginase (GRN 428), and lactase (GRN 510). The genotype of strain DS38556 appears to be similar to that of ISO-502, and this strain has been used to produce acid prolyl endopeptidase (GRN 832) and phospholipase A1 (GRN 857).

Another strain used for protein production is *A. niger* AGME9. This strain was developed from *A. niger* ATCC 14916 through UV mutagenesis to enhance glucoamylase production. *A. niger* AGME9 was used by Danisco to produce glucose oxidase (GRN 1054), which is used in the production of baked goods, cheese, and mayonnaise.

According to the January 2026 inventory of microorganisms notified to EFSA for QPS assessment, approximately 70 notifications involved *A. niger* (Table 4). Among these, approximately 50 are related to protein production for food applications, whereas the remaining are associated with feed additives. These notifications should not be interpreted as indicating that all corresponding strains or applications were included in the QPS list or recommended for QPS status. Compared with GRAS Notices, EFSA notification records often provide limited information regarding production-strain lineages and manufacturing processes. Therefore, direct equivalence between strains described in EFSA records and those described in GRAS Notices cannot be established without supporting documentation. In addition to Novozymes and DSM Food Specialties, another company reported to use *A. niger* for protein production is Shin Nihon Chemical Co., Ltd. This company produces enzymes such as catalase, 3-phytase, amylase, and lipase using *A. niger* [46-48]. A notable difference is that, unlike the two aforementioned companies, these enzymes are produced using non-GMO strains.[Table T5]

Further examination of the EFSA notification inventory indicates that *A. niger* is also frequently used as production organism for feed additives (Table 6). For instance, Andrés Pintaluba S.A. produces the product Amylofeed^®^ using endo-1,3(4)-β-glucanase, endo-1,4-β-xylanase, and α-amylase. In Amylofeed^®^, endo-1,3(4)-β-glucanase and endo-1,4-β-xylanase are produced by *A. niger* NRRL 25541, whereas α-amylase is produced by *A. oryzae* ATCC 66222 [49, 50]. Similarly, BASF SE produces several animal feed additives under the Natuphos^®^ brand. Among these, the 6-phytase included in Natuphos^®^ E is produced by *A. niger* DSM 25770 [51, 52], whereas Natugrain^®^ TS contains endo-1,4-β-xylanase produced by *A. niger* CBS 109.713 and endo-1,4-β-glucanase produced by *A. niger* DSM 18404 [53, 54].

### Aspergillus oryzae

Selected strains of *A. oryzae* have a long history of use in traditional fermented foods and have also been described as production organisms in GRAS Notices [55]. In particular, *A. oryzae* RIB40 is regarded as the national fungus (“kokkin”) in Japan and is widely used in the Japanese traditional food industry [56]. Similar to *A. niger*, *A. oryzae* produces considerable amounts of enzymes, such as amylases and proteases, and is extensively used during the primary fermentation stage of traditional fermented foods [38]. For instance, during soy sauce fermentation, *A. oryzae* secretes various enzymes that degrade macromolecules, such as proteins and carbohydrates in soybeans, into peptides or oligosaccharides. These compounds are subsequently utilized by yeasts during the secondary fermentation process, enabling the production of alcohols, volatile organic compounds (VOCs), and various other metabolites. Additionally, *A. oryzae* produces various secondary metabolites that contribute to flavor development as well as antimicrobial activity. Several studies have suggested that the growth of *A. oryzae* in food may inhibit the growth of toxigenic *A. flavus*, indicating a potential functional role in enhancing food safety.

Unlike *A. niger*, *A. oryzae* has not been widely reported to produce major mycotoxins [45]. However, concerns remain regarding compounds such as cyclopiazonic acid (CPA) and 3-nitropropionic acid (NPA). Although *A. oryzae* does not produce aflatoxins, some strains retain partial aflatoxin biosynthetic gene clusters in their genomes, raising concerns regarding their safety. Therefore, companies producing industrial enzymes attempt to improve host strains by removing certain secondary metabolite pathways to enhance safety. Such genetic modifications should be accompanied by analytical confirmation that the relevant metabolites are absent from the production process and final product.

**Fig. 3:**
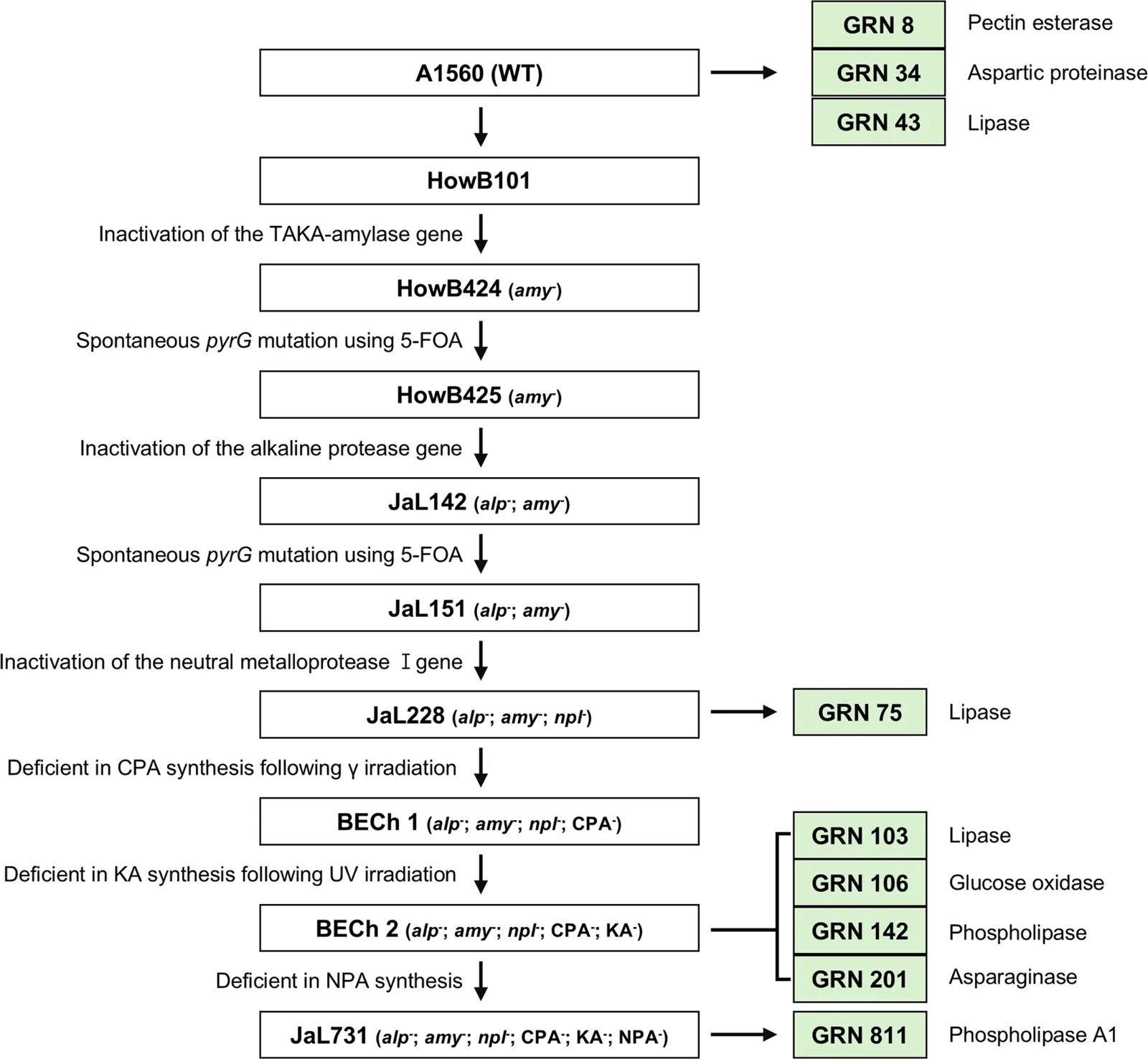
Lineage of *A. oryzae* strains derived from the A1560 strain.

Our review of the GRAS notification inventory identified 20 GRAS Notices involving *A. oryzae* production strains or their derived substances, of which 10 were submitted by Novo Nordisk or Novozymes North America Inc. (Table 5). The strains most frequently utilized are *A. oryzae* IFO 4177 (A1560) and its derived A1560 lineage strains. *A. oryzae* A1560 is known as a wild-type “koji mold” strain and has been widely used industrially because of its high protein secretion ability [57]. Based on the A1560 strain, additional strain improvement was achieved through genetic engineering and mutagenesis to generate recipient host strains such as Jal228 and BECh2 (Fig. 3). *A. oryzae* A1560 has been used to produce enzymes such as pectin esterase (GRN 8), aspartic proteinase (mucopepsin; GRN 34), and lipase (GRN 43). The wild-type A1560 strain was subsequently improved through various strain engineering approaches. HowB771 was developed from A1560 through the deletion of the TAKA-amylase gene and was used for the production of *Myceliophthora thermophila* laccase (GRN 122). Particularly, A1560-derived strains were extensively engineered using *pyrG* auxotrophic marker recycling systems to enable efficient protein production. The *pyrG* mutant strain HowB101 was first generated from wild-type A1560 through homologous recombination. The *pyrG* auxotrophic marker was then used to construct HowB424, a TAKA-amylase gene (amy^−^) deletion mutant. Subsequently, 5-fluoro-orotic acid (5-FOA) treatment was applied to induce mutations in *pyrG*, generating HowB425, in which the *pyrG* marker could be recycled. Using this sequential engineering strategy, the alkaline protease (*alp*) gene and natural metalloprotease I (*NpI*) gene were deleted, ultimately resulting in the construction of strain JaL228. This strain was then engineered to express a lipase gene from *Fusarium oxysporum* to produce triacylglycerol lipase (GRN 75).

Although JaL228 was useful for high-efficiency protein production, it retained the ability to produce certain secondary metabolites characteristic of *A. oryzae*. To overcome this limitation, sequential gamma irradiation and UV irradiation were used to eliminate cyclopiazonic acid and kojic acid synthesis. The resulting strain, BECh2, was subsequently used to produce four proteins: synthetic lipase (GRN 103), *A. niger* glucose oxidase (GRN 106), phospholipase A1 from *Fusarium venenatum* (GRN 142), and *A. oryzae* asparaginase (GRN 201). Additionally, strains derived from BECh2 were further engineered to eliminate the potentially toxic metabolite 3-nitropropionic acid (3-NPA), yielding recipient strains such as JaL731. These strains were used to express phospholipase A1 from *Valsaria rubricosa*. Furthermore, the French precision fermentation company Verley (formerly Bon Vivant) developed the strain *A. oryzae* TFB-CLEO75TA (GRN 1241), which was derived from improvements of the JaL731 lineage and is used to produce bovine β-lactoglobulin (*Bos taurus* β-lactoglobulin).

GRAS Notices involving the *A. oryzae* A1560 lineage cover a broad range of industrial applications, although several other strains have also been employed. Some of these are wild-type, non-GMO strains used to produce enzymes and minerals. For instance, Cura Global Health uses the *A. oryzae* KOJI strain for mineral production (GRN 829) [58]. Godo Shusei uses the *A. oryzae* GD-FAL (GODO-FAL) strain for the production of lactase (GRN 1039) [59], and Mitsubishi Chemical Corporation uses *A. oryzae* NBRC 110971 for the production of tannase used in beverages, jelly products, and botanical extracts. Nanjing Bestzyme Bio-Engineering Co., Ltd. developed strain M3-114 through UV mutagenesis and CRISPR/Cas9 genome editing (GRN 1207). In this strain, the *amyA* and *amyB* genes (encoding α-amylases), *alp* (encoding alkaline protease), *Np1* (encoding neutral metalloprotease), *cpaA* (associated with cyclopiazonic acid biosynthesis), *kojR* (associated with kojic acid biosynthesis), and *agsB* (encoding α-1,3-glucan synthase) were edited. These modifications were intended to eliminate the potential production of harmful secondary metabolites and enhance protein production efficiency. The resulting strain, *A. oryzae* 90402, was subsequently used to produce brazzein, which is used as a food sweetener. The corresponding GRAS conclusion concerns brazzein produced under the specified manufacturing process and intended conditions of use, rather than conferring GRAS status on strain 90402 or *A. oryzae* generally.

According to the inventory of microorganisms notified to EFSA for QPS assessment, approximately 30 notifications involved *A. oryzae*. These notifications can be divided into two main categories: protein production and feed additives (Table 4). Novozymes uses *A. oryzae* production strains to manufacture enzymes including xylanase, α-amylase, lipase, asparaginase, and carboxypeptidase D. However, the publicly available notification data are insufficient to establish whether strains designated with the “NZYM” prefix are identical to, or closely related to, strains in the A1560 or BECh2 lineages. Any such relationship should therefore be stated only when supported by the corresponding regulatory dossier or published strain information (Table 6). These enzymes, including α-amylase, 6-phytase, and endo-1,4-β-xylanase, are used for the degradation of polysaccharides, phytates (a phosphorus-containing compound), and non-starch polysaccharides (NSPs), such as xylan, present in feed, thereby improving feed efficiency as well as nutrient digestibility in livestock. Various companies formulate and market products optimized for cattle, pigs, poultry, and other animals using combinations of these enzymes. For instance, the RONOZYME^®^ product line produced by DSM Nutritional Products Ltd. contains 6-phytase or endo-1,4-β-xylanase produced by *A. oryzae*. In addition to proteins produced by *A. oryzae*, the fungal strain can also be directly used as a feed additive. For instance, *A. oryzae* KCTC 10258BP, when combined with *Bacillus subtilis* KCCM 10673P and applied to soybeans, has been reported to effectively reduce raffinose family oligosaccharides and trypsin inhibitors present in soybeans [60, 61].

In conclusion, *A. oryzae* is one of the most important fungi used in the traditional production of fermented foods. Advances in genetic engineering approaches, including CRISPR-based technologies, enable further optimization of this species to enhance production efficiency and to eliminate potentially toxic secondary metabolites. Therefore, *A. oryzae* is expected to continue playing an important role in the enzyme and food industries.

## Future Perspectives

**Fig. 4:**
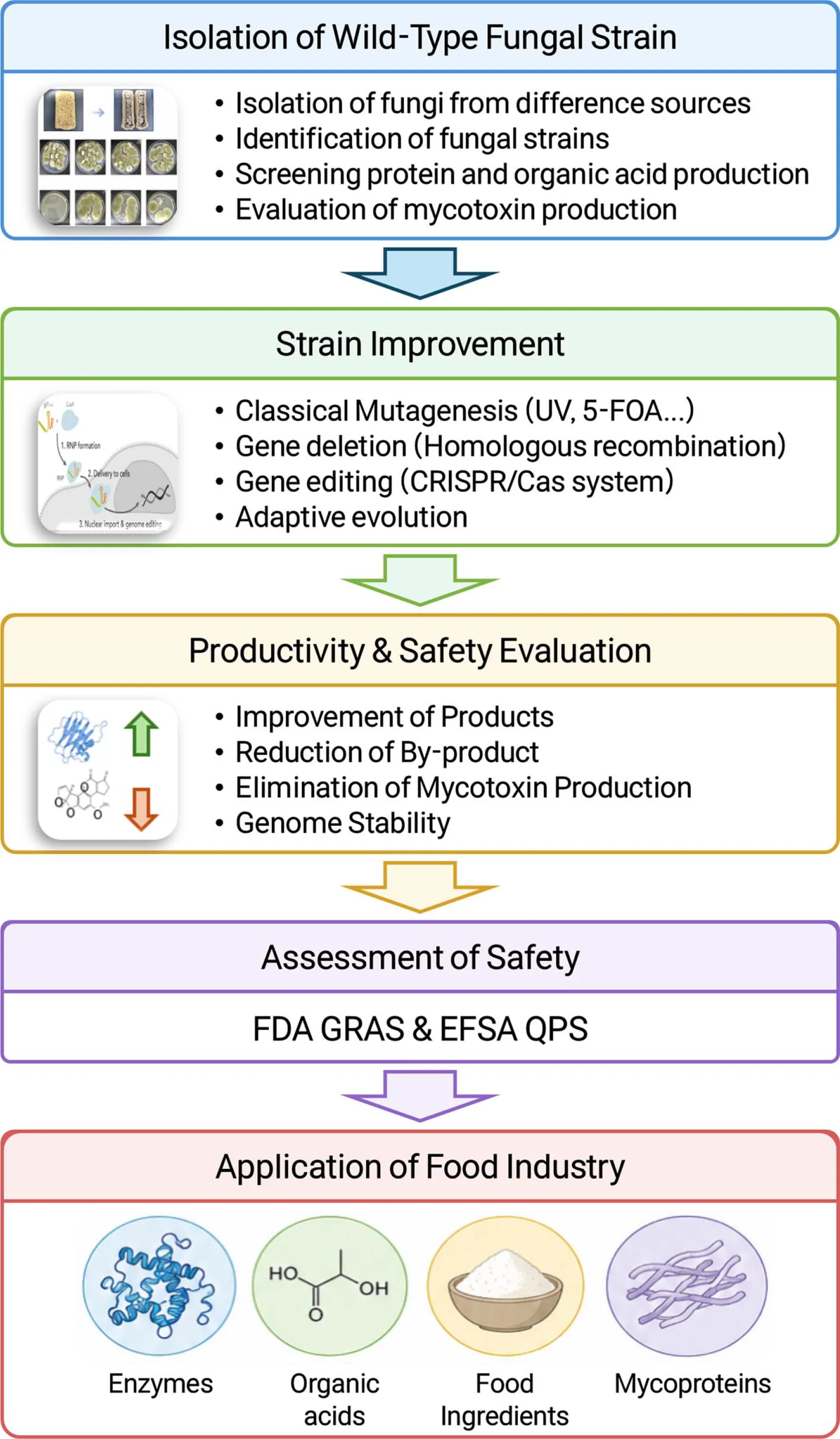
Overview of industrial fungal strain development for food applications. Wild-type fungal strains are isolated and screened for desirable traits, followed by strain improvement using classical and modern genetic engineering approaches. Improved strains are evaluated for productivity, product quality, genome stability, and safety, including elimination of undesirable metabolites. After regulatory assessment under frameworks such as FDA GRAS and EFSA QPS, qualified strains are utilized for the commercial production of food enzymes, organic acids, food ingredients, mycoproteins, and other fermentation products.

Currently, filamentous fungi and fungal-derived enzymes are utilized in a wide range of applications. As summarized in Fig. 4, the development of industrial fungal strains for food applications begins with the isolation, identification, and screening of promising wild-type strains. Strains isolated from natural environments have been developed through classical mutagenesis using methods such as UV irradiation, chemical mutagenesis, or other conventional strain improvement approaches. Additionally, strain improvement has been further advanced through homologous recombination-based genetic engineering. The main objectives of strain improvement include: 1) editing or deleting genes linked to endogenous protease genes and native amylase genes to improve recombinant protein production, 2) removing genes associated with mycotoxin production, 3) enhancing the secretion system to increase protein secretion, and 4) improving overall protein production. To enhance protein expression systems, various strategies can be employed, such as replacing the 5′ UTR or promoter region, inserting signal peptides, optimizing codon usage, using synthetic genes, and introducing multi-copy expression systems.

In filamentous fungi, multiplex genome editing has traditionally been challenging because of the limited availability of selectable markers. To overcome this limitation, marker recycling strategies have been employed to enable sequential deletion of multiple genes, but these approaches are time-consuming. The development of the CRISPR–Cas9 system has largely overcome these limitations by enabling efficient multiplex genome editing [62-64]. For example, recent studies have demonstrated multiplex CRISPR–Cas9 approaches in which a CRISPR multiplexing strategy in which multiple guide RNAs are used to simultaneously target several genes, thereby significantly accelerating industrial strain improvement. This strategy also facilitates the simultaneous disruption of multiple harmful secondary metabolite biosynthetic gene clusters, reducing the potential for mycotoxin production and ultimately improving the safety of fungal production strains. Furthermore, the use of CRISPR–Cas9 ribonucleoprotein (RNP) complexes provides an additional advantage because genome editing can be achieved without introducing foreign DNA, such as antibiotic resistance markers or Cas9 expression cassettes, into the fungal genome. Consequently, RNP-mediated genome editing has emerged as a promising strategy to reduce the introduction of exogenous DNA into production strains, which may simplify regulatory evaluation in some jurisdictions.

In addition, advances in artificial intelligence (AI) combined with multi-omics datasets are expected to further accelerate the development of industrial fungal strains. AI-driven analyses can predict candidate genes associated with enhanced production of enzymes, organic acids, and other valuable metabolites. AI-based approaches can also be applied to guide RNA design, enzyme engineering, promoter optimization, and signal peptide design to improve gene expression, enzyme activity, and protein secretion [65]. The integration of these AI-derived predictions with CRISPR–Cas9 genome editing will enable more precise and efficient strain engineering for enhanced productivity [66]. Moreover, AI-assisted metabolic pathway analysis can identify genetic targets for reducing by-product formation, thereby increasing product yield and purity. Likewise, transcriptomic, proteomic, and metabolomic analyses of secondary metabolite biosynthetic pathways can facilitate the identification of genes involved in mycotoxin biosynthesis and support rational engineering strategies to reduce the production of undesirable metabolites, further supporting the development of production strains with improved safety profiles and their final products. In addition, these emerging technologies including CRISPR genome editing and AI-assisted genome analysis are expected to facilitate the development and evaluation of safer fungal production strains by providing more comprehensive genetic and functional characterization.

These strains are improved to enhance product yield, reduce by-product formation, and eliminate mycotoxin production. Following evaluations of their productivity and safety, the improved strains may undergo regulatory evaluation under applicable frameworks, such as the FDA GRAS Notices or the EFSA QPS approach, depending on the intended production and jurisdiction and subsequently be used for the production of enzymes, organic acids, mycoproteins, and other valuable products.

Although gene editing or genetic engineering can be used to generate improved fungal strains by modifying or deleting genes involved in mycotoxin biosynthesis or harmful by-product formation, such genetic modifications alone do not fully demonstrate the safety of the resulting strains or their final products. Therefore, additional safety evaluations are required to support regulatory assessment. First, metabolomic and proteomic analyses should be performed to confirm the absence of mycotoxins, harmful metabolites, and undesirable by-products. Second, the safety of the final products should be evaluated using appropriate chemicals, *in vitro*, and, where necessary, *in vivo* toxicity assessments. Third, the allergenic potential of both the production organism and the final product should be evaluated. Collectively, these data would provide essential evidence for safety evaluations conducted under regulatory frameworks such as the FDA GRAS Notices and the EFSA QPS approach, together with other product-specific regulatory requirements.

In conclusion, the integration of artificial intelligence, CRISPR-Cas9 technology, and synthetic biology can improve the efficiency and accuracy of genome editing while facilitating the development of production strains with improved productivity and safety profiles. These advances will accelerate the development of filamentous fungus-based foods and food ingredients, thereby opening new avenues for the future food industry.

## Figures and Tables

**Table 1:** Fungal species included in the EFSA QPS list.

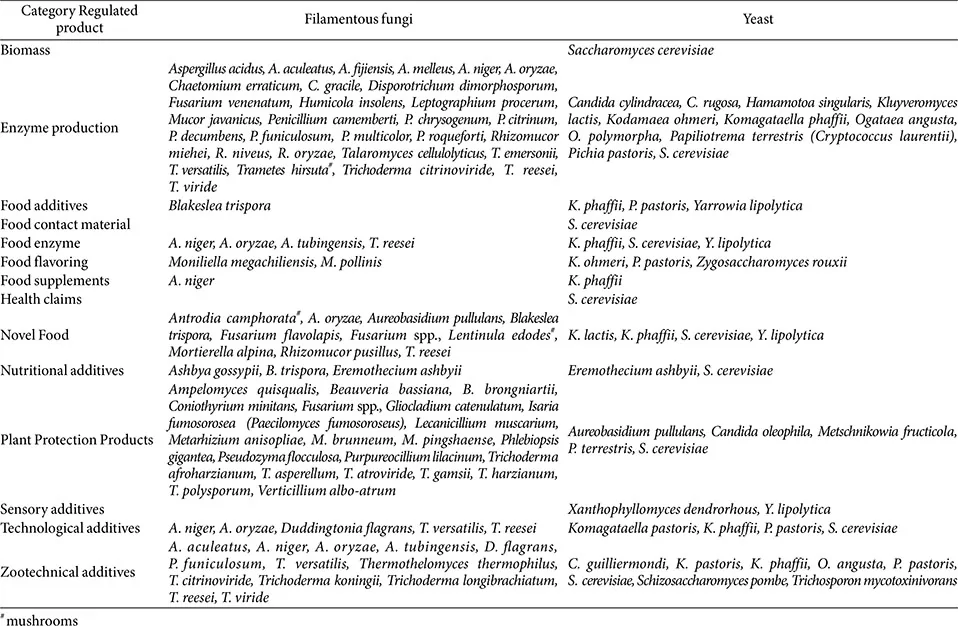

**Table 2:** Fungal species included in the GRAS list.

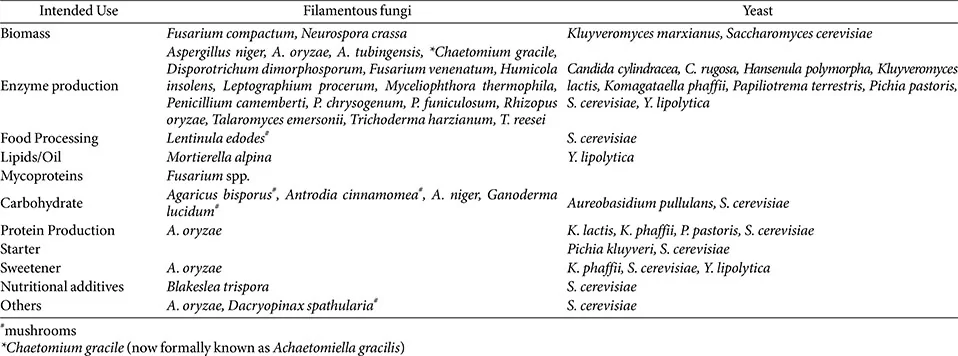

**Table 3:** Industrial applications of *A. niger* strains presented in GRAS notifications.

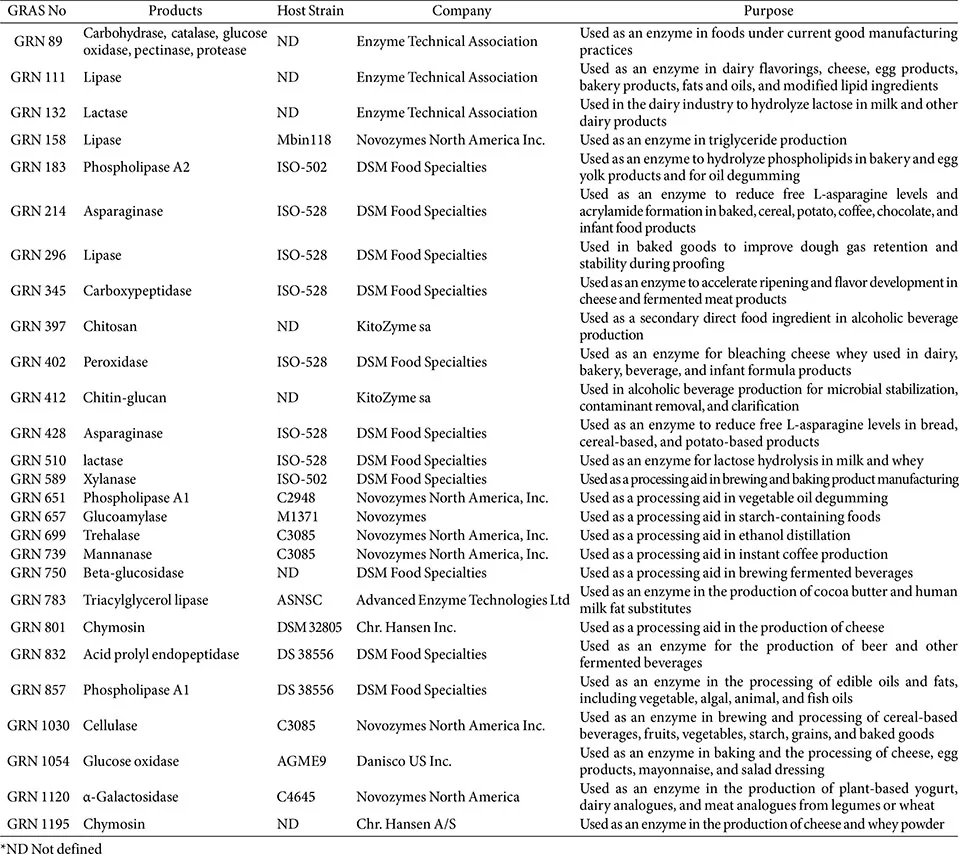

**Table 4:** Industrial applications of *A. niger* and *A. oryzae* in the EFSA QPS list.

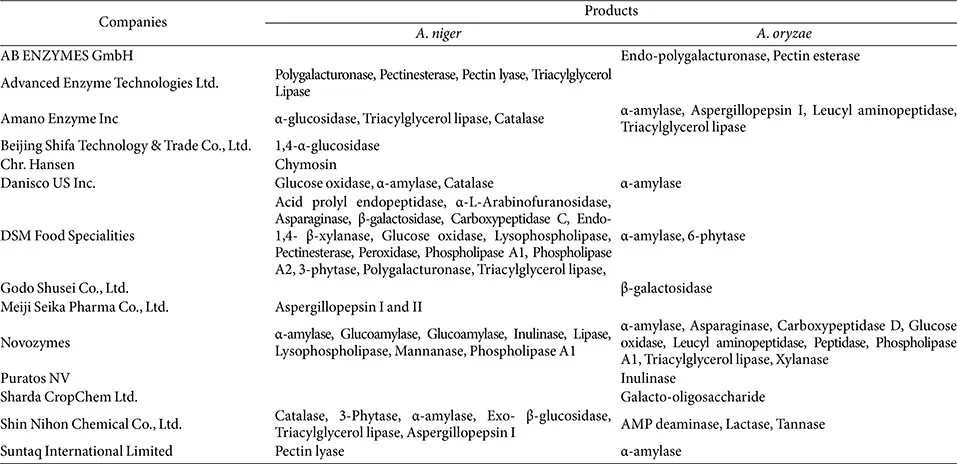

**Table 5 T5:** Industrial applications of *A. oryzae* strains presented in GRAS notifications.

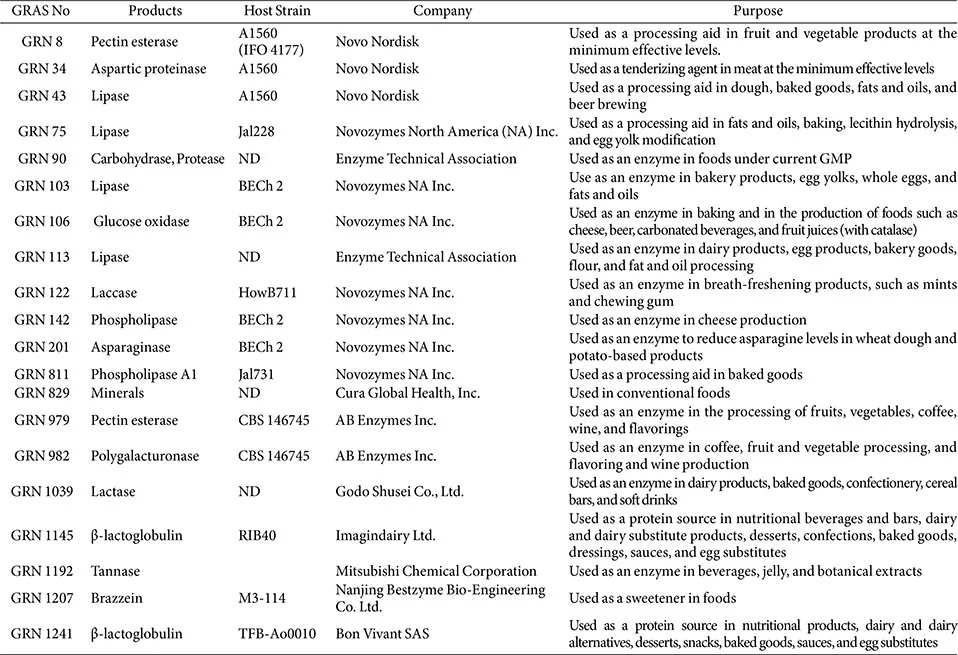

**Table 6:** Feed additives using *A. niger* and *A. oryzae* in the QPS system.